# Supplementary material for: “Two Cultures in Favor of a Dying Patient”: Experiences of Health Care Professionals Providing Snakebite Care to Indigenous Peoples in the Brazilian Amazon
Source: Toxins (Basel). 2023 Mar 3;15(3):194. doi: 10.3390/toxins15030194 (PMC10051728; doi:10.3390/toxins15030194)
Supplement: Supplementary file 1 [file toxins-15-00194-s001.zip › toxins-2219643-supplementary.pdf]

toxins

MDPI

Article

"Two Cultures in Favor of a Dying Patient": Experiences of Health Care Professionals Providing Snakebite Care to Indigenous Peoples in the Brazilian Amazon

Felipe Murta, Eleanor Strand, Altair Seabra de Farias, Felipe Rocha, Alícia Cacau Santos, Evellyn Antonieta Tomé Rondon, Ana Paula Silva de Oliveira, Hiran Satiro Souza da Gama, Yasmim Vieira Rocha, Gisele dos Santos Rocha, Mena Ferreira, Vinícius Azevedo Machado, Marcus Lacerda, Manuela Pucca, Felipe Cerni, João Ricardo Nickenig Vissoci, Anna Tupetz, Charles J. Gerardo, Ana Maria Moura-da-Silva, Fan Hui Wen, Jacqueline Sachett and Wuelton Monteiro

Consolidated criteria for reporting qualitative studies (COREQ): 32-item checklist

No. Item	Guide questions/description	Reported on Page #
	**Domain 1: Research team and reflexivity**	
	Personal Characteristics	
1. Interviewer/facilitator	*Which author/s conducted the interview or focus group?*	Pages 557-571
2. Credentials	*What were the researcher's credentials?*	Pages 557-571
3. Occupation	*What was their occupation at the time of the study?*	Pages 557-571
4. Gender	*Was the researcher male or female?*	Pages 557-571
5. Experience and training	*What experience or training did the researcher have?*	Pages 557-571
Relationship with participants		Page 7
6. Relationship established	*Was a relationship established prior to study commencement?*	Pages 569-571
7. Participant knowledge of the interviewer	*What did the participants know about the researcher? (e.g. personal goals, reasons for doing the research).*	Pages 569-571
8. Interviewer characteristics	*What characteristics were reported about the inter viewer/facilitator? e.g. Bias, assumptions, reasons and interests in the research topic.*	Pages 557-571
	**Domain 2: study design**	
	Theoretical framework	
9. Methodological orientation and Theory	*What methodological orientation was stated to underpin the study? e.g. grounded theory, discourse analysis, ethnography, phenomenology, content analysis*	Pages 594-604
Participant selection		
10. Sampling	*How were participants selected? e.g. purposive, convenience, consecutive, snowball*	Pages 573-578
11. Method of approach	*How were participants approached? e.g. face-to-face, telephone, mail, email*	Pages 573-578

12. Sample size	*How many participants were in the study?*	Pages 573-590
13. Non-participation	*How many people refused to participate or dropped out? Reasons?*	Pages 88-90
Setting		
14. Setting of data collection	*Where was the data collected? e.g. home, clinic, workplace*	Pages 525-537
15. Presence of non-participants	*Was anyone else present besides the participants and researchers?*	Pages 557-571
16. Description of sample	*What are the important characteristics of the sample? e.g. demographic data, date*	Pages 80-93
Data collection		
17. Interview guide	*Were questions, prompts, guides provided by the authors? Was it pilot tested?*	Page 591
18. Repeat interviews	*Were repeat inter views carried out? If yes, how many?*	N/A
19. Audio/visual recording	*Did the research use audio or visual recording to collect the data?*	Pages 580-590
20. Field notes	*Were field notes made during and/or after the inter view or focus group?*	Pages 580-590
21. Duration	*What was the duration of the inter views or focus group?*	Pages 580-590
22. Data saturation	*Was data saturation discussed?*	Pages 580-590
23. Transcripts returned	*Were transcripts returned to participants for comment and/or correction?*	N/A
	**Domain 3: analysis and findings**	
Data analysis		
24. Number of data coders	*How many data coders coded the data?*	Pages 593-604
25. Description of the coding tree	*Did authors provide a description of the coding tree?*	Pages 593-604
26. Derivation of themes	*Were themes identified in advance or derived from the data?*	Pages 593-604
27. Software	*What software, if applicable, was used to manage the data?*	Pages 593-595
28. Participant checking	*Did participants provide feedback on the findings?*	N/A

Developed from:

Tong, A.; Sainsbury, P.; Craig, J. Consolidated criteria for reporting qualitative research (COREQ): A 32-item checklist for interviews and focus groups. *Int. J. Qual. Health Care* **2007**, *19*, 349–357.
